# Interspecific Divergence of Two *Sinalliaria* (Brassicaceae) Species in Eastern China

**DOI:** 10.3389/fpls.2018.00077

**Published:** 2018-01-31

**Authors:** Lei Zhang, Tingting Zeng, Huan Hu, Liqiang Fan, Honglei Zheng, Quanjun Hu

**Affiliations:** Key Laboratory for Bio-Resource and Eco-Environment of Ministry of Education, College of Life Science, Sichuan University, Chengdu, China

**Keywords:** *Sinalliaria*, common garden, morphological delimitation, reproductive isolation, genetic divergence

## Abstract

How endemic species originated in eastern Asia has interested botanists for a long time. In this study, we combined experimental and computational modeling approaches to examine the morphological and genetic divergence and reproductive isolation of two tentative species of *Sinalliaria* (Brassicaceae) endemic to eastern China, *S. limprichtiana* and *S. grandifolia*. Most of the examined morphological characters (including hairs of leaf blades and stems, corolla length and width, and flower stalk length) were well-delineated between two species at the same ploidy level, and there was clear evidence of reproductive isolation between them (mainly due to post-pollination barriers) in the common garden environment. There were also strong and consistent divergences in the population genetic data. Coalescent simulations based on sequence variation of the nuclear genes suggest that interspecific divergence began during the Pleistocene when the climate oscillated in eastern Asia. Gene flow between two species appears to have been very limited and asymmetrical. Our results suggested that both species are well-differentiated and that the fast divergence between them might have been together shaped by both stochastic processes and habitat selection pressures.

## Introduction

For several decades, biogeographic researchers have considered the reasons for the high species diversity of plants and numerous endemics in eastern Asia, especially compared with eastern North America, which has a similar size, climate, and floristic components (e.g., Tiffney, 1985; Axelrod et al., 1998; Qian and Ricklefs, 2000). The high diversity has been attributed to a combination of two major processes (Liu, 1988; Qian and Ricklefs, 2000; Qian et al., 2003). First, numerous ancient genera and species may have persisted due to geographical and ecological heterogeneity that could buffered the organisms from small-scale extinctions caused by the climate oscillations in eastern Asia, in conjunction with the lack of Pleistocene glaciation and subsequent large-scale extinctions. Second, many young species (for example, four *Dysosma* species, *D. pleiantha, D. versipellis, D. difformis*, and *D. majoensis*) have originated through fast divergence probably driven by Pleistocene climate oscillations in conjunction with physiographic heterogeneity (Ikeda et al., 2012; Han et al., 2016; Wang et al., 2017). Both hypotheses have been partly tested based on biogeographic analyses of a few genera and phylogeographic examinations of some widely distributed species (see summaries by Qiu et al., 2011; Liu et al., 2012). The direct evidence for such hypotheses should derive from the times and processes of the well-delimitated sister species that may have occurred during the Pleistocene (Zanella et al., 2016; Wang et al., 2017). Although Ikeda et al. (2012), Han et al. (2016), and Wang et al. (2017) have revealed the Pleistocene origins of a pair of endemic alpine species, two coastal wild radish lineages and four understory herbs based on population genetic data, it remains unknown whether these species or lineages were morphologically differentiated with stable distinct characters and reproductively isolated as “good species” based on the integrate species concept (Liu, 2016).

**Figure 1:**
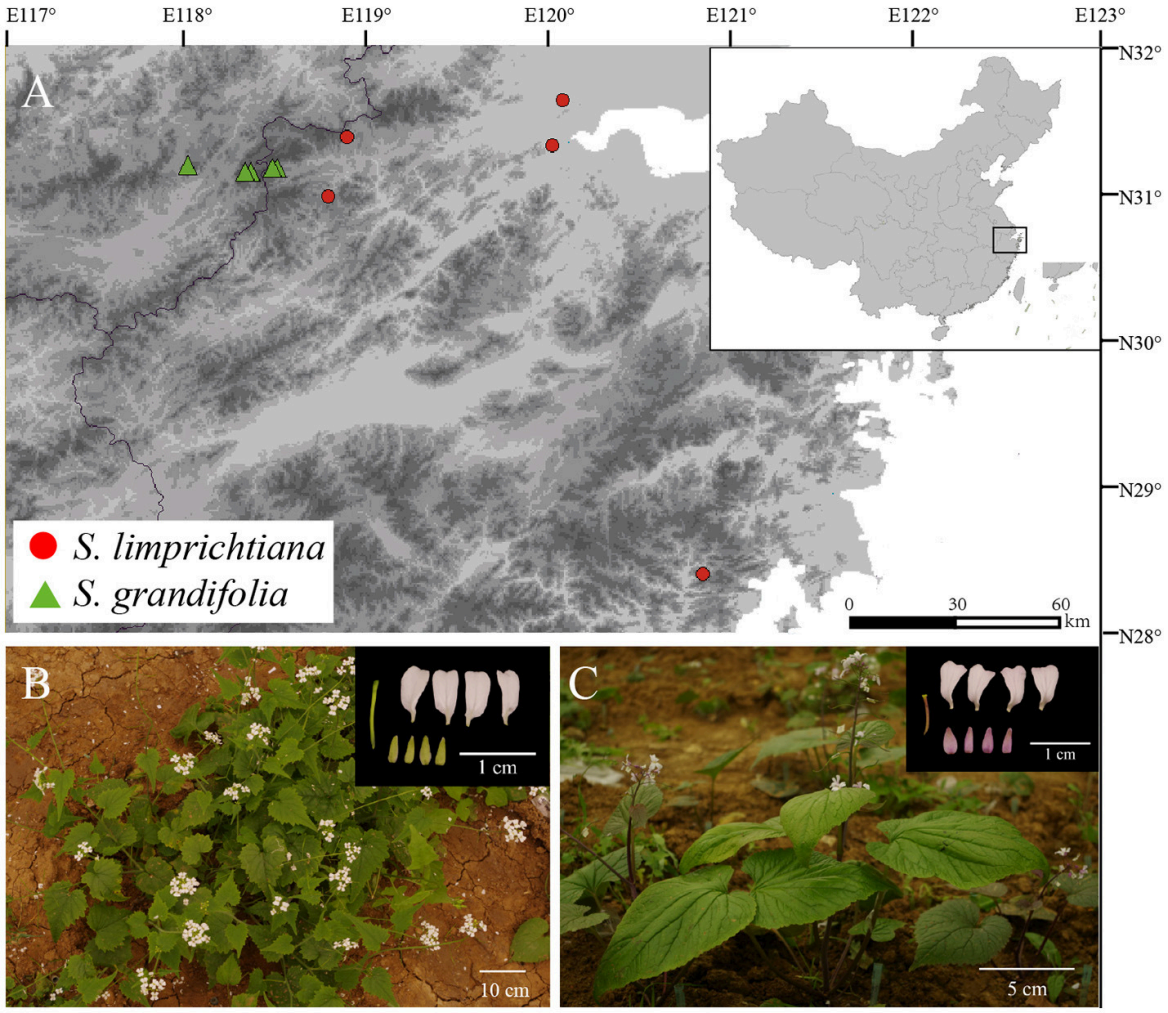
**(A)** Distributions of the two *Sinalliaria* species showing locations of the populations of *S. limprichtiana* (red circles) and *S. grandifolia* (green triangles) sampled in this study. **(B)** Photograph of *S. limprichtiana*. **(C)** Photograph of *S. grandifolia*.

Here we focus on the two only known annual species of the genus *Sinalliaria, S. limprichtiana*, and *S. grandifolia* (Brassicaceae). Both species are endemic to eastern Asia (Zhou et al., 2014; Hu et al., 2015), and narrowly distributed in warm-temperate evergreen forests or forest edges in eastern China (Figure 1) (Al-Shehbaz and Guang, 2000; Zhou et al., 2014). These two taxa were treated as two subspecies by some authors (Zhou et al., 2014) and they were found to have clear genetic distinctions based on sequence variations of the chloroplast DNAs and the limited number of the sampled individuals for each species (Hu et al., 2015, 2016). Nevertheless, whether morphological differentiation remains stable and reproductive isolation has been well-established between them still need to be confirmed. Moreover, the divergence time and demographic history of two species remains to be elucidated using population genetic data at multiple nuclear loci.

In the present study, we first examined the ploidy level, morphological differentiation, and strength of two species' pre- and post-pollination reproductive isolation in a common garden when ecological factors were totally excluded. Then we measured the genetic divergence at the population level between them, according to sequence variation of both 12 Simple Sequence Repeat (SSR) loci and nine unlinked, low-copy nuclear genes. Finally, we quantified changes in their effective population sizes and amounts of gene flow during their speciation and established whether the interspecific divergence was related to Pleistocene climate changes with the relatively young age.

## Materials and methods

### Common garden experiments

To assess phenotypic differences between two species, we used three *S. limprichtiana* populations and two *S. grandifolia* populations (Figure 1, Table S1). For each population, we selected five individuals. For each individual, we used 10 mature seeds collected from the field. The seeds were kept at 4°C for 3 weeks and then sown in Petri dishes containing water-soaked filter paper at 25°C for 3–7 days. Five to 10 resulting seedlings for each field individual were transplanted to 42 mm-diameter pots and placed in a growth chamber at 25°C during vegetative growth for 1 week. After forming three leaves, all seedlings were moved from pots to an experimental field in the Sichuan University campus in October 2015. We measured germination rates each month. All seedlings started to flower in March and set fruit in April 2016.

### Determination of ploidy level by flow cytometry

We estimated the amounts of nuclear DNA in each species' genome following the protocol described by Dolezel and Bartos (2005). Briefly, we isolated the nuclei from fresh leaves of each three individuals, from three *S. limprichtiana* populations and two *S. grandifolia* populations mentioned above, using Otto I solution, and subsequently suspended them with a mixture of Otto I and Otto II solution (1:4) in phosphate/citric acid buffer, pH 7.3. After 30 min staining with propidium iodide, nuclear fluorescence was measured using a Partec CA-II flow cytometer, with *Vigna radiata* (2C = 1.40 pg DNA and 1 pg DNA = 9.78 × 10^8^ base pairs) as a calibration standard (Dolezel and Bartos, 2005). We used the coefficient of variation (CV = standard deviation/peak mean × 100%) of DNA peaks to quantify the precision of individual measurements with the value of CV < 3% calculated by FlowJo software.

### Morphological differentiation

**Figure 2:**
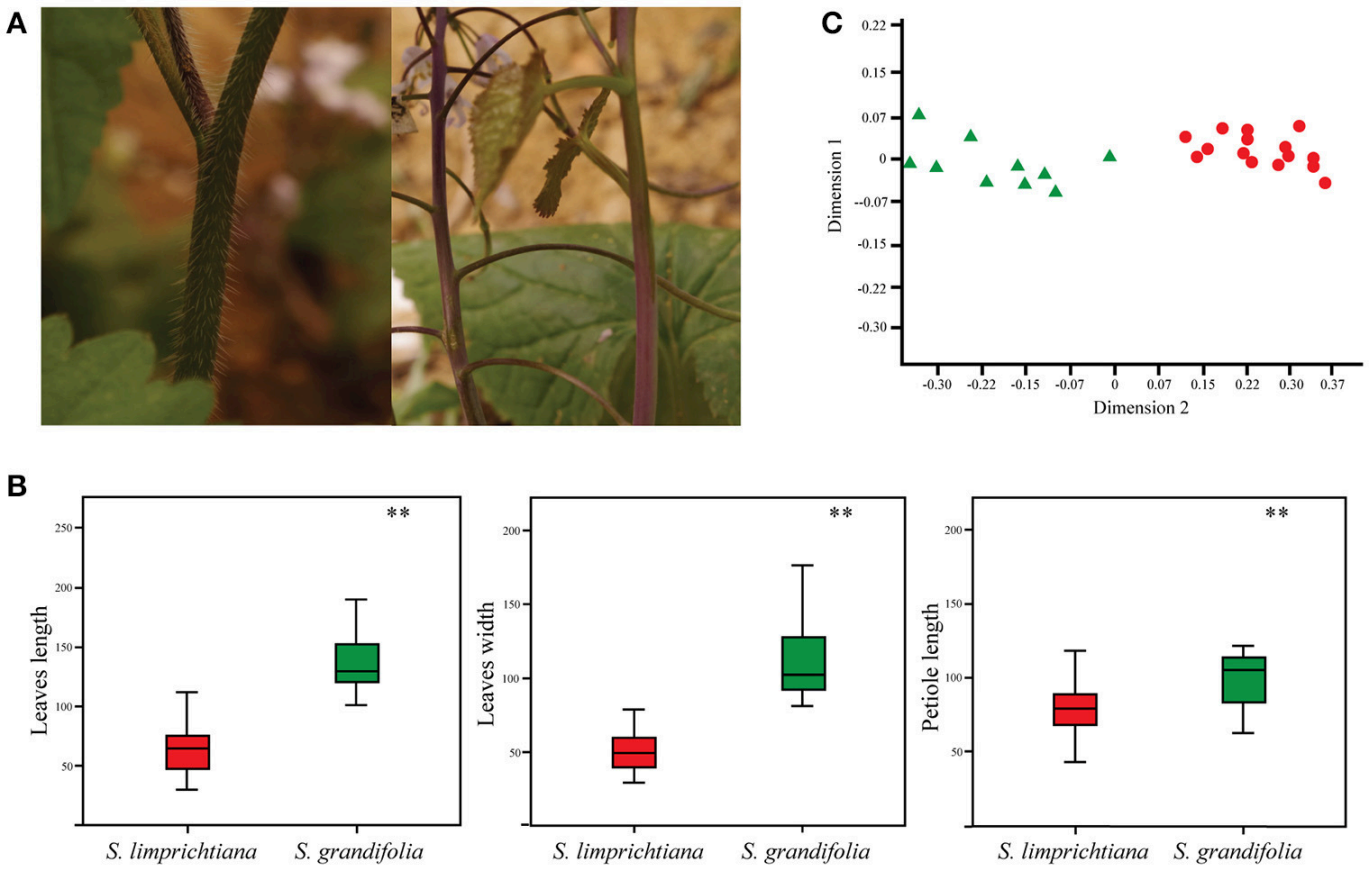
**(A)** Hair presence/absence in *S. limprichtiana* (left) and *S. grandifolia* (right). **(B)** Contrasting sizes of the basal leaves of *S. limprichtiana* and *S. grandifolia* (Significance level, ^**^P < 0.01). **(C)** Morphological clusters based on Principal Coordinate Analysis of 15 traits of *S. limprichtiana* (red circles) and *S. grandifolia* (green triangles).

To minimize the morphological variations caused by ecological factors, we randomly selected five descendants cultivated from each of the five individuals of each population of *S. limprichtiana* and *S. grandifolia* in the common garden at the full flowering stage. Firstly, we recorded the presence or absence of hairs on their leaf blades and stems. Then we examined and/or measured the characters of five flowers and sets of three basal leaves, central-stem leaves, and upper-stem leaves produced by each individual. These traits included six floral characters (corolla length and width, flower stalk length, calyx length, number of inflorescence branches, and number of flowers; Figure S3), and nine leaf characters (length, width, and petiole lengths of each set of leaves) (Figure 2B, Figure S2). We used SPSS version 23.0 software to calculate means and standard deviations of these variables, and apply one-way analysis of variance (ANOVA) to assess the variability of each measured trait (Day and Quinn, 1989; Schürch et al., 2000), deeming differences to be significant if *p* < 0.01. Finally, we used principal co-ordinate analysis (PCoA; Bitner-Mathé and Klaczko, 1999), implemented in the Multivariate Statistics Package (MVSP) version 3.2 (http://www.kovcomp.com/), to assess the distinctiveness of two species, based on all examined and measured traits.

### Pre- and post-pollination reproductive isolation

We evaluated the strength of pre-pollination isolation between the species by examining the overlap of their flowering periods, defined as the time (about 56 days) when an individual had at least one open flower, with pollen and/or a wet receptive stigma. We daily observed and recorded the number of these “active” flowers on five individuals cultivated from seeds collected from each of five individuals from each population.

We also evaluated the strength of the species' post-pollination reproductive isolation by comparing fruit set arising from inter-specific crossing, intra-specific (but inter-population) crossing, and spontaneous self-pollination. For all crossing experiments, we removed anthers before flowering and then bagged flowers. At the flowering stage, pollen was removed from the anthers of the designated “male” flowers and transferred to the receptive stigmatic cavities of the designated “female” flowers using a tooth pick with marker tags. All artificially pollinated flowers were re-bagged. The flowers assigned to the self-pollination treatment were continuously bagged and not otherwise manipulated. For each individual, only one flower was chosen for each of three above-mentioned experiments. A month after the pollination treatment we collected siliques and recorded the fruit set (defined as the number of full fruits divided by sum of fruits and undeveloped fruits). Differences in fruit set between the crossing types (and selfing treatment) were evaluated by ANOVA, followed by Student's two-tailed *t*-test for paired comparisons, with SPSS version 23.0 software, deeming differences to be significant if *p* < 0.05.

### Extraction of total DNA

Using a modified CTAB method (Doyle, 1987), we extracted total genomic DNA from ~100 mg portions of dry leaf material collected from five and four natural populations of *S. grandifolia* and *S. limprichtiana*, respectively (Table S2). One population of *S. limprichtiana* is distributed far away from the other four (Figure 1), indicating a wide disjunction. However, there is no specimen record and our field explorations also failed to find its occurrence in other regions.

### “SSR” primer development

First, we extracted RNA from fresh leaves of one individual of each species using TRIZOL reagent (Sigma Aldrich). Then we sequenced them to develop primers to amplify target sequences. Trinity (http://trinityrnaseq.sourceforge.net/) was used to assemble the RNA-Seq data, which retrieves large fractions of transcripts, especially abundant transcripts (Grabherr et al., 2011). To avoid redundancy in the datasets, we used CD-HIT (http://weizhong-lab.ucsd.edu/cd-hit) to obtain unigene sequences. The Microsatellite Identification Tool (MISA, http://pgrc.ipk-gatersleben.de/misa/) was used to identify microsatellites in the unigenes. Twelve microsatellite loci containing three-nucleotide motifs with at least five repeats were selected (Table 1). Using Primer version 3.0 software, primers with complementary sequences to flanking regions of these SSRs were then designed, varying in length from 20 to 23 base pairs (bp; close to the reportedly optimal length of 20 bp), with GC contents varying between 45 and 65% (close to the reported optimal 50%) (Qi et al., 2016). We also developed primers (Table 1) for nine low-copy nuclear genes with Primer version 6.0 software, using homologous EST genes of two species (Wang et al., 2011). Functional annotation of the corresponding genes was performed by BLAST searches of the NCBI database.

**Table 1:** Characteristics of 12 EST-SSR and nine nuclear low-copy gene primer pairs validated in a survey of Sinalliaria populations.

**Loci**	**Forward primer**	**Reverse primer**	**Length**	**Product size**	**GC%**	**Tm (°C)**	**Repeat Motif**	**Multiplex panel**
**SSR**								
*SSR_1*	TCAGGCTTATGTGGATGCTG	CAAGCTTGCATTTGTGTGCT	20	240	60	55	(CTA)7	P3
*SSR_2*	CATGAATATGCAAGAGCGGA	CCAAGGACAGGCTTCTTCAG	20	182	60	55	(GAA)5	P3
*SSR_3*	GAGCTCGTTACCGTTTAGCG	CGCTCATCAAAATGCAGAAA	20	275	60	55	(TGA)8	P3
*SSR_4*	AGCTTCTTCCATTAACGCCA	TCATGATCTGTTCACTCGCC	20	207	60	55	(CAT)8	P3
*SSR_5*	AAACCGCCTCCGATTCTATT	GGTTCGAATTTTCATCGGAA	20	219	60	55	(CCG)5	P3
*SSR_6*	CCGGAATTAATATCTAAACCTGTAA	TCTTTCTCAGCCTTTTCCCA	25	202	59	55	(AGA)6	P3
*SSR_7*	CAAGCAGAGATCGTGGATGA	ACTGGCGAAGCTCGTGTTAT	20	154	60	55	(GCA)5	P3
*SSR_8*	CCCACCAACTCCACCATAAA	CGTCATCAAGACGTCGAAGA	20	237	60	55	(TCT)8	P3
*SSR_9*	AAAATGGAGATGGCGACAAG	ATTCCGGTTCGGCTAGAGAT	20	233	60	55	(CAT)8	P3
*SSR_10*	GCCATTCTTCCGTCAGTCTC	GCCTCAGCGATAATCTCAGC	20	224	60	55	(TCT)6	P3
*SSR_11*	CTGTCGGAGACTCACTTCCC	GACTAGTTGGTCGGCTCTCG	20	271	60	55	(ACA)6	P3
*SSR_12*	CGTCCGAGAGAAAGCAGAAG	ACCAGAAGTGACGAGAGCGT	20	272	60	55	(AGA)6	P3
LOW-COPY NUCLEAR GENES^*^								
*SINTR2771_02*	GATTGAGTTCACGCAACAT	CTCCGCATCTGAAGTCATA	19	579	42.1	55		
*H3 gene 2*	CTTGCTACTAAGGCTGCTC	ACGCTCACCTCTTATACGA	19	356	52.6	55		
*LOC106423940*	AAGACGACTCTATCCAACTC	GGAATCCGACTCGCTATC	20	253	45	55		
*EUTSA_v10020616 mg*	TAGAGACGAAGTTGTGAGAG	AATAGATGGAGTACGAGACAG	20	261	45	55		
*EUTSA_v10010760 mg*	TCCACATCCGCCATCAACA	TATCACAACCACCGAAGAACTC	19	295	52.6	55		
*EUTSA_v10007525 mg*	CCCAGAGAAAGGAGGATTG	CCATCTTGTACTGTGATACG	19	271	52.6	55		
*MEF9*	ATGCACCAAATCGTCAACAA	TTCGTCCCATAGTCCCATGT	20	528	50	55		
*SINATR1498_34*	TGGTCATGAATCAGCAGGAA	TCCAGGGAAGCTGAAAGAGA	20	622	47.3	55		
*SINATR2110_55*	CATAAGCCTTCCTGCCTGTT	TGTCAGCCTCTCCTTGTATCT	20	638	50.0	55		

* *Gene description based on best hit to NCBI nucleotide database. SINTR2771_02, Early light-induced protein 2, chloroplastic-like; H3 gene 2, Histone H3.3; LOC106423940, Uncharacterized mRNA; EUTSA_v10020616 mg, Uncharacterized mRNA*.

*EUTSA_v10010760 mg, Monothiol glutaredoxin-S14, chloroplastic-like; EUTSA_v10007525 mg, Uncharacterized mRNA; MEF9, Pentatricopeptide repeat-containing, mitochondrial-like; SINATR1498_34, Uncharacterized mRNA; SINATR2110_55, DNA replication complex GINS protein PSF3-like*.

### Genotyping polymorphisms of SSR loci

In total, 38 individuals representing four populations of *S. limprichtiana* and 36 individuals representing five *S. grandifolia* populations were genotyped using the 12 pairs of EST-SSR primers (Table 1). After a 5-min denaturation at 95°C, PCR was performed for 35–36 cycles (95°C for 45 s, 55°C for 40 s, and 72°C for 80 s), with a 7–10 min final extension at 72°C. The resulting PCR products were differentiated and genotyped using an ABI 3730 xL automated sequencer.

### Sequencing low-copy nuclear genes

We amplified nine low-copy nuclear genes (Table 1) in 19 individuals representing four *S. limprichtiana* populations and 20 individuals representing four *S. grandifolia* populations. Amplifications were done by initial denaturation (94°C, 5 min), 35–36 cycles of denaturation (94°C, 30 s), annealing (55°C, 45 s) and extension (72°C, 50–60 s), and final extension (72°C, 7–10 min). The resulting products were purified using a TIANquick Midi Purification Kit (Tiangen Biotech, Beijing, China), then sequenced with primers covering the whole PCR segments using an ABI Prism Bigdye™ Terminator Cycle Sequencing Ready Reaction Kit, and visualized using an ABI 3730 xL sequencer. Sequences were aligned using Clustal X (Thompson et al., 1997) with default parameters and curated manually. The boundaries of each dataset were delimited by comparison with sequences of the sister species. The obtained sequences have been deposited in GenBank under accession numbers KY689753-KY689824.

### Genetic diversity and divergence at SSR loci

We calculated genetic diversity parameters for the 12 SSR loci in both species, including information index (*I*), expected heterozygosity (*H*_e_), observed heterozygosity (*H*_o_), unbiased expected heterozygosity (*uH*), fixation index (*F*) values and *F*-statistics using GenAlex version 6.5 (Peakall and Smouse, 2012). We also examined the genetic variance within and among populations and species by analysis of molecular variance (AMOVA), using ARLEQUIN version 3.5.1.2 (Excoffier and Lischer, 2010).

We examined the population structure of two species, based on variation at the 12 SSR loci, using STRUCTURE version 2.3 software (Hubisz et al., 2009). Twenty independent runs with the number of clusters (*K*) varied from 1 to 20, in each case with 500,000 iterations and a 50,000 burn-in period (based on the initial comparison) using prior population information and an admixture model assuming correlated allele frequencies among clusters were applied to infer population structure according to Pritchard et al. (2000). Δ*K* values based on the rate of change in the log probability of data between successive *K* values were estimated and the most likely cluster was chosen (Evanno et al., 2005). Then, we used PCoA to explore the internal structure of the presence/absence matrix for the entire data set using GenAlEx version 6.5 (Krzanowski, 2000). In PCoA multivariate statistics are used to depict the genetic structure, without relying on the population genetics assumptions underlying STRUCTURE (Engelhardt and Stephens, 2010).

### Genetic divergence at nine nuclear low-copy genes

We used PHASE version 2.1.1 to determine the haplotype phase of the directly sequenced PCR products for each nuclear locus for each individual (Stephens et al., 2001). We aligned the multiple sequences for each gene by MEGA version 6.0, then constructed haplotype networks, representing unique alleles separated by mutational steps, using the median-joining method (Bandelt et al., 1999) implemented in NETWORK version 5.0 (http://www.fluxus-technology.com/sharenet.htm). Next, we applied DnaSP version 5.10 to calculate basic parameters (Librado and Rozas, 2009) for each gene, including the number of synonymous substitutions, the number of haplotypes (*N*_h_), haplotype diversity (*H*_e_), minimum number of recombination events (*R*_m_), average number of nucleotide polymorphisms per site (π) between two sequences in a sample (Nei, 1987), and θ_w_ = 4*N*_e_μ, where *N*_e_ is the effective population size and μ is the mutation rate per site per generation) based on the number of segregating sites (Watterson, 1975). We also calculated Tajima's *D* (Tajima, 1989), Fu and Li's D^*^ and F^*^ statistics, using DnaSP version 5.10 (Fu and Li, 1993), to test the neutrality of each gene's mutations. Moreover, we constructed their phylogenetic relationships using the neighbor-joining approach with 500 bootstrap replicates, as implemented in MEGA version 6.0. The same as the SSRs dataset, we also used ARLEQUIN version 3.5.1.2 and STRUCTURE version 2.3 to evaluate the partitioning of genetic diveristy and population structure based on the nuclear genes.

### Coalescence-based analyses of divergence times

We examined the species' speciation history using the following demographic parameters derived from analyses of the low-copy nuclear genes: population-split time (*T*), bidirectional migration rate *m* (*m*_GL_ and *m*_LG_ represent the migration rate from the *S. limprichtiana* to the *S. grandifolia* and from the *S. grandifolia* to the *S. limprichtiana*, respectively), and effective sizes of the ancestral (θ_A_) and descendant populations (θ_L_ and θ_G_). We calculated all parameters using the IM model implemented in the IMa2 program. The IM model assumes no selection, no recombination within loci, no substantial population structure, and random mating in ancestral and descendent populations (Hey and Nielsen, 2004, 2007). These assumptions are unlikely to be completely met in real populations. However, parameter estimates of the IM model have been found to be robust to high levels of population structure and recombination (Carstens and Knowles, 2007). We used the program to estimate parameters of the IM model, and assessed the posterior probability densities for model parameters using Markov Chain Monte Carlo (MCMC) methods (Hey and Nielsen, 2007; Hey, 2010). After several preliminary runs to optimize prior boundaries for the six primary demographic parameters (*T, m*_LG_, *m*_GL_, θ_A_, θ_L_, and θ_G_), we set the length of the burn-in period of 1 × 10^5^ iterations, followed by 1 × 10^6^ MCMC steps and Metropolis-coupling of 120 independent heated chains. We assessed the MCMC mixing properties based on the effective sample sizes (ESS), swapping rates between successive MCMC chains, and trend-line plots of the parameters. We repeated the well-mixed runs to obtain reproducible results when three independent runs produced similar posterior distributions (Ikeda et al., 2009; Wang et al., 2013).

All demographic parameters have to be scaled using the geometric mean of the mutation rate per year per locus during IMa2 calculations, so selection of appropriate mutation rates is crucial for estimates of almost all parameters (Strasburg and Rieseberg, 2010). The chalcone synthase (*CHS*) gene in Brassicaceae reportedly has a synonymous mutation rate (μ_*CHS*_) of 1.5 × 10^−8^ substitutions per site per year (Koch et al., 2000). Therefore, following Ikeda et al. (2009) and Wang et al. (2013), we estimated the mutation rate (μ) at silent sites of each locus using the equation μ = μ_*CHS*_ × *K*_Total_*/K*_S_ × *L*, where *K*_Total_*/K*_S_ is the ratio of the average total genetic divergence to the average synonymous genetic divergence, and *L* is the length of the focal locus. The estimated geometric average of *K*_Total_*/K*_S_ over all loci was 0.8353. We therefore used the derived geometric mean of 5.2947 × 10^−6^ substitutions per locus per year (yr) to scale the demographic parameters.

## Results

### Interspecific differentiation in genome sizes and morphology

We estimated genome size and ploidy level of each species by flow cytometry (FCM). As shown in Table S3, the flow cytometry results yielded 2C DNA values for the three sampled individuals from different populations of *S. limprichtiana* ranging from 1.54 to 1.70 pg (mean and standard deviation: 1.604 ± 0.09 pg), and corresponding values for *S. grandifolia* are 1.55 to 1.71 pg (1.647 ± 0.08 pg) (Table S3). Both species are at the same ploidy level with very similar genome sizes (Figure S1).

There were no clear differences in overall germination and survival rates between two species, but we observed clear morphological differences. First, hairs were present on calyxes, leaf blades and stems of *S. limprichtiana*, but not *S. grandifolia* (Figure 2A). Second, *S. limprichtiana* has shorter and thinner basal and stem leaves than *S. grandifolia* (Figure 2B, Figure S2). Third, *S. limprichtiana* produces more flowers and inflorescence branches than *S. grandifolia* (Figures S3E,F). Finally, *S. limprichtiana* has shorter flower corollas than *S. grandifolia* (Figure S3A). PCoA analyses of the 15 examined traits clearly distinguished the species (Figure 2C).

### Pre- and post-pollination reproductive isolation

**Figure 3:**
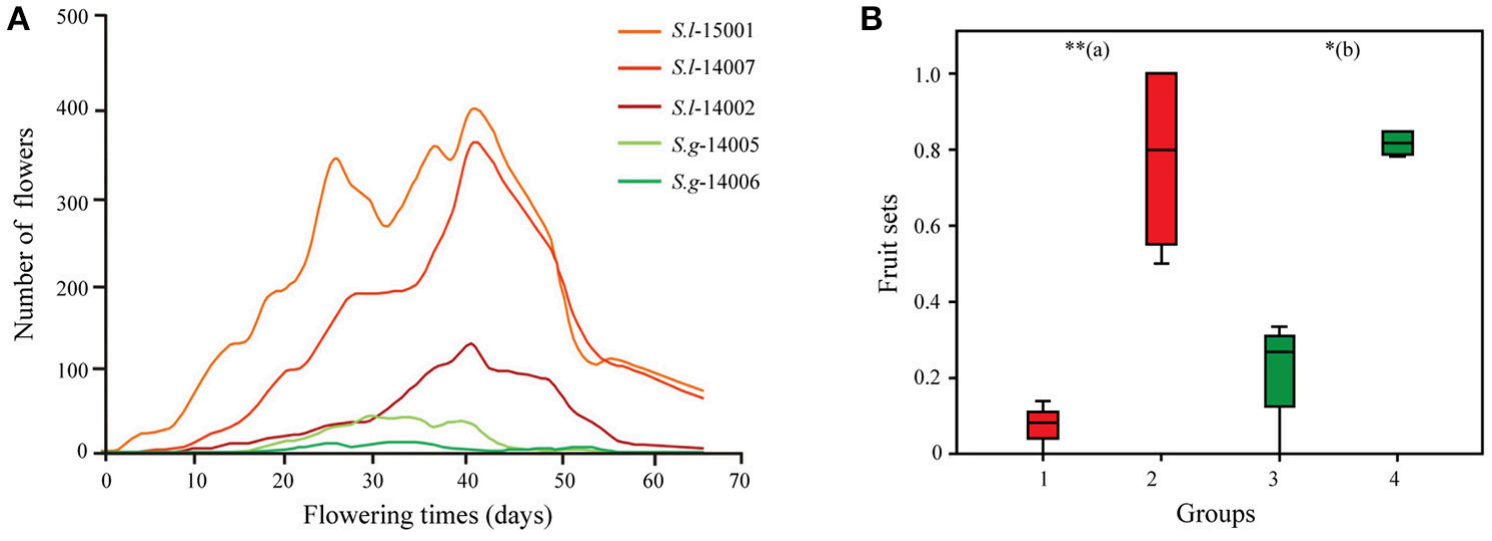
**(A)** Flowering periods of cultivated individuals of the two *Sinalliaria* species in the common garden (red lines for individuals representing different populations of *S. limprichtiana* and green lines for individuals representing different populations of *S. grandifolia*). **(B)** Fruit sets of monitored female parents of two species (red and green for *S. limprichtiana* and *S. grandifolia* respectively), pollinated interspecifically (groups 1 and 3) or by members of different populations of the same species (groups 2 and 4). (a) indicates results of tests of differences between groups 1 and 2, and (b) indicates results of tests of differences between groups 3 and 4. Significance level: ^**^0.001 ≤ *P* < 0.01; ^*^0.01 ≤ *P* < 0.05.

The flowering times of the artificially constructed populations of each species in the common garden experiment (intended to minimize the effects of environmental factors) strongly overlapped (Figure 3A), although *S. limprichtiana* started and stopped flowering somewhat earlier than *S. grandifolia*. All bagged flowers for selfing experiments failed to set fruit. Interspecific crosses resulted in significantly lower (*p* < 0.001) fruit set (8.1–33.3%) than crosses between populations of the same species (50.0–100%) in the reciprocal crossing experiments (Figure 3B).

### Genetic diversity and differentiation at SSR loci

**Figure 4:**
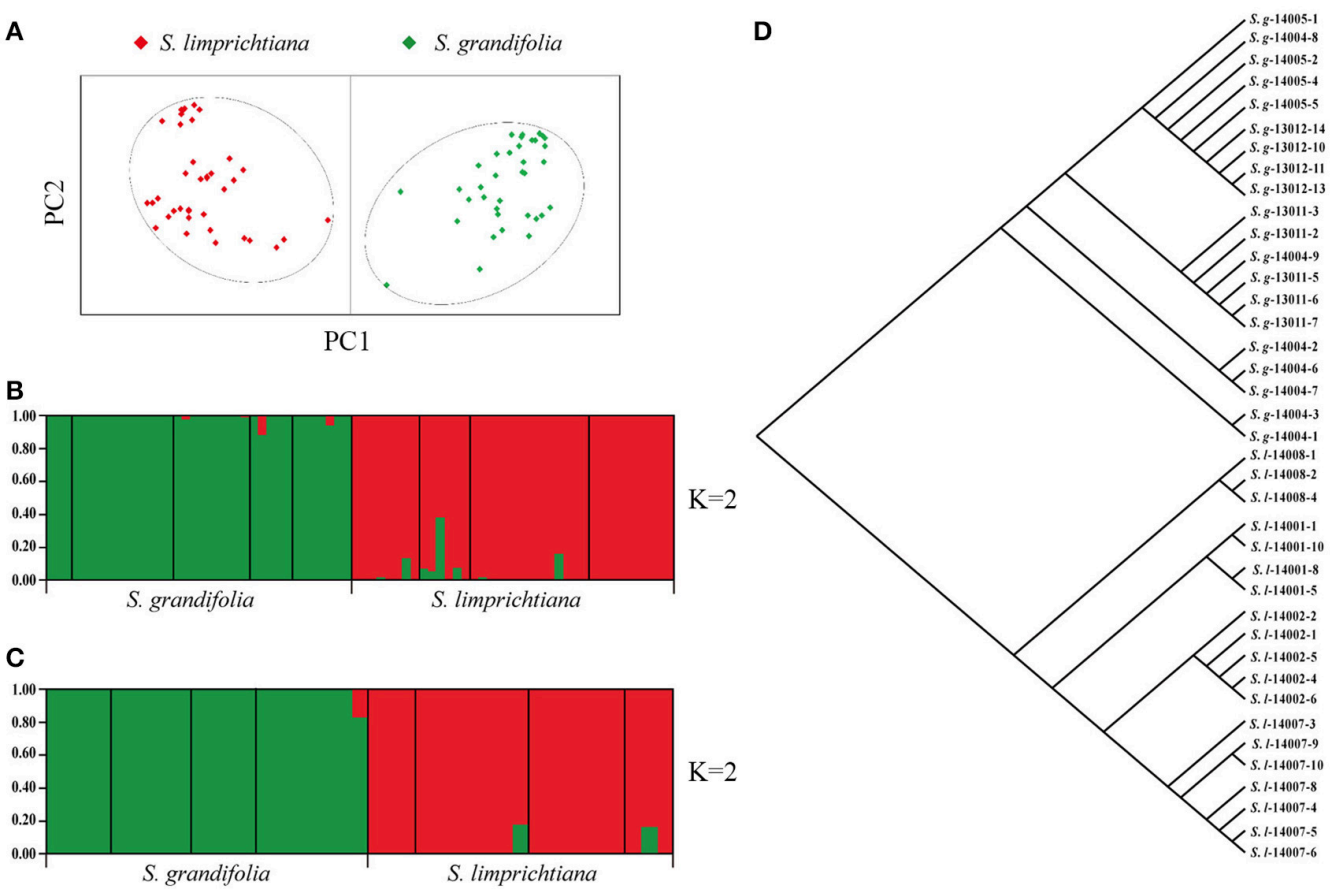
**(A)** Results of Principal Coordinate Analysis of genetic distances among nine populations of two species, based on 12 SSR loci (red and green spots for individuals of *S. limprichtiana* and *S. grandifolia*, respectively). **(B,C)**. Two species' genetic structure, based on 12 SSR loci and nine low-copy nuclear genes, respectively. **(D)** Neighbor-joining tree derived from phylogenetic analysis of *S. limprichtiana* and *S. grandifolia* (*S.l*. and *S.g*. respectively) based on nine low-copy nuclear genes.

Averaged across all nine populations, observed heterozygosity (Ho) per locus was 0.378, and expected heterozygosity (He) was 0.414 (Table S4). The fixation index averaged across all loci (average *F*_ST_ = 0.372; Table S4) indicated a pronounced level of genetic differentiation among populations (Bleeker and Hurka, 2001; Helenurm, 2003). PCoA of data acquired from all individuals again identified two well-delimited groups (Figure 4A), which were supported by STUCTURE analyses, with each group representing a different species (best K = 2, Delta K = 283.38; Figure 4B, Figure S4). AMOVA analyses also revealed clear differentiation between both species and intraspecific populations (*F*_ST_ = 0.461, *p* < 0.001; Table 2).

**Table 2:** AMOVA analyses of genetic partitions between and within *S. limprichtiana* and *S. grandifolia* for 12 SSR loci and nine nuclear low-copy genes.

**Source of variation**	***df***		***SS***		***VC***		***V%***		***F*****-statistic**	
	**SSR**	**Low-copy**	**SSR**	**Low-copy**	**SSR**	**Low-copy**	**SSR**	**Low-copy**	**SSR**	**Low-copy**
**All samples**										
Among species	1	1	65.846	418.261	0.71237	19.23659	24.77	67.66	*F*_SC_ = 0.28475^*^	*F*_SC_ = 0.87446^*^
Among populations	7	6	78.637	235.961	0.60387	8.03932	21	28.28	*F*_ST_ = 0.46180^*^	*F*_ST_ = 0.95940^*^
Within populations	65	31	113.125	35.779	0.181	35.779	6.29	4.06	*F*_CT_ = 0.24773^*^	*F*_CT_ = 0.67663
Total	147	38	359.608	690	2.87562	28.43006				
***S. limprichtiana***										
Among populations	3	3	26.611	217.897	0.42356	15.45513	28.12	15.45513		
Within populations	72	15	77.955	23.893	1.08271	23.893	71.88	9.34	*F*_ST_ = 0.2812^*^	*F*_ST_ = 0.90657^*^
Total	75	18	104.566	241.789	1.50627	241.789				
***S. grandifolia***										
Among populations	4	3	35.442	18.064	0.56184	1.07726	32.81	59.19		
Within populations	67	16	77.1	11.886	1.15075	11.886	67.19	40.81	*F*_ST_ = 0.32806^*^	*F*_ST_ = 0.59186^*^
Total	71	19	112.542	29.95	1.71258	1.82012				

df, degrees of freedom; SS, sum of squares; VC, variance components; V%, percent variation; F_ST_, differentiation among species; F_SC_, differentiation among populations within species; F_CT_, differentiation within populations;

* *P < 0.01, 1000 permutations*.

### Genetic diversity and divergence of nine low-copy nuclear genes

**Figure 5:**
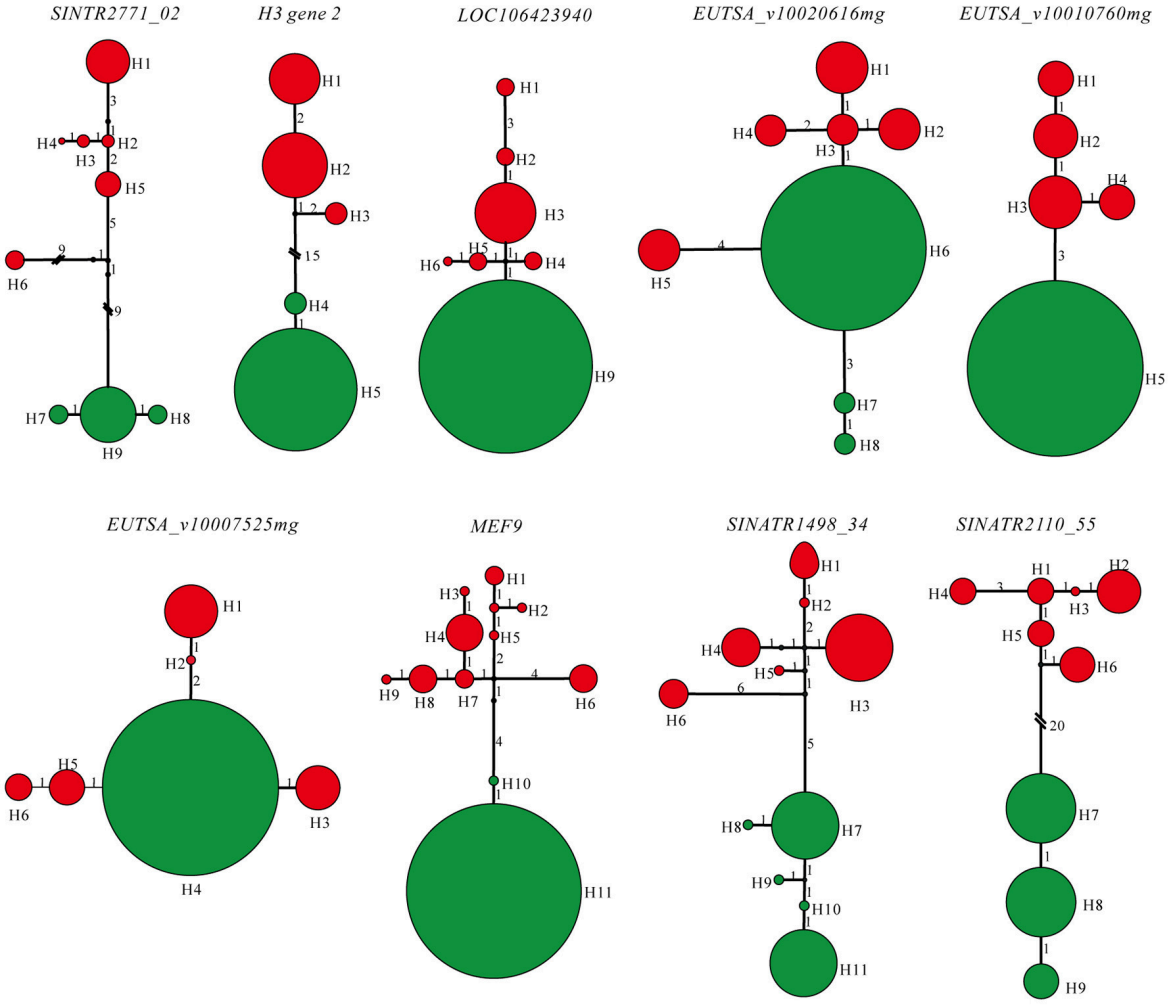
Genealogies of the nine low-copy nuclear loci. Colors in the pie chart indicate the haplotype origin; red, *S. limprichtiana*; green, *S. grandifolia*. The size of the pie is proportional to the haplotype frequency found in two species. Marked branch lengths indicate the mutation steps.

Lengths of the nine sequenced nuclear genes extracted from individuals representing populations of two species ranged from 253 to 638 bp, with a total concatenated length of 3803 bp, excluding gaps and missing data. More haplotypes of each gene were detected in *S. limprichtiana* than in *S. grandifolia* (Figure 5). Haplotype networks for each gene suggested that two species have completely diverged, and private SNPs of both species were detected in genealogies of seven of the nine loci (Figure 5). Diversity in *S. limprichtiana* is higher than in *S. grandifolia* (Table 3). Tajima's *D*, Fu and Li's D^*^ and F^*^ neutrality indicators varied widely across the nine genes (Table 3). No significant Tajima's *D* values were obtained for any of the nine genes in either species (*p* < 0.05), but significant D^*^ values were obtained for three loci (*SINTR2771_02, MEF9* and *SINATR1498_34*) and significant F^*^ values for one gene (*SINTR2771_02*) in *S. limprichtiana*. Since the three tests did not provide consistent significant indications of deviation from neutrality in any of the nine loci (Table 3), all of the genes were used for subsequent analyses.

**Table 3:** Summary of the nucleotide polymorphisms and neutrality tests of nine nuclear genes for *S. limprichtiana* and *S. grandifolia*.

								**Neutrality test**		
**Species**	**Gene**	***R*_m_**	***S***	**θ_w_**	**π**	***H*_d_**	***N*_h_**	**Tajima *D'***	D^*^ test	F^*^ test
***S. limprichtiana***										
	*SINTR2771_02*	1	20	0.00988	0.01222	0.8129	6	0.91014	1.57747^**^	1.60538^*^
	*H3 gene 2*	0	5	0.00402	0.00513	0.6491	3	0.85651	1.19239	1.26649
	*LOC106423940*	1	7	0.00792	0.00878	0.7895	6	0.52031	1.30356	1.20079
	*EUTSA_v10020616 mg*	0	7	0.00767	0.00910	0.8363	5	0.61913	1.30356	1.28295
	*EUTSA_v10010760 mg*	0	3	0.00291	0.00416	0.7836	4	1.17129	1.01467	1.21433
	*EUTSA_v10007525 mg*	0	6	0.00633	0.00967	0.8012	5	1.70313	1.25359	1.59057
	*MEF9*	2	11	0.00596	0.00753	0.9181	10	0.94758	1.43728^*^	1.50155
	*SINATR1498_34*	1	14	0.00644	0.00731	0.8070	6	0.50385	1.49918^**^	1.40513
	*SINATR2110_55*	0	8	0.00359	0.80700	0.8538	6	0.91403	1.34523	1.41352
	Average	0.56	9	0.00608	0.09677	0.8057	5.67			
***S. grandifolia***										
	*SINTR2771_02*	0	2	0.00097	0.00093	0.4895	3	0.11187	0.86615	0.69109
	*H3 gene 2*	0	1	0.0008	0.00076	0.2684	2	0.0861	0.64952	0.52031
	*LOC106423940*	Null	0	0	0	0	1	Null	Null	Null
	*EUTSA_v10020616 mg*	0	4	0.00432	0.0046	0.3579	3	0.18621	1.10821	0.98261
	*EUTSA_v10010760 mg*	Null	0	0	0	0	1	Null	Null	Null
	*EUTSA_v10007525 mg*	Null	0	0	0	0	1	Null	Null	Null
	*MEF9*	0	1	0.00053	0.00019	0.1	2	−1.16439	−1.53959	−1.53959
	*SINATR1498_34*	1	4	0.00181	0.003	0.7474	5	1.90245	1.10821	1.52994
	*SINATR2110_55*	0	2	0.00089	0.00133	0.674	3	1.15776	0.86615	1.08407

R_m_, estimate of minimum number of recombination events; S, number of polymorphic sites; θ_w_, Watterson's estimator of θ per base pair; π, nucleotide diversity; N_h_, number of haplotypes; H_d_, haplotype diversity; Tajima D' and Fu & Li's D^*^ and F^*^ (Fu and Li, 1993). Significant level:

* 0.01 ≤ P < 0.05;

** *0.001 ≤ P < 0.01*.

Levels of nucleotide polymorphism (θ_w_ and π) differed strongly among the nine genes (Table 3). For each gene, the average nucleotide diversity was higher in *S. limprichtiana* than in *S. grandifolia* (Table 3). The minimum number of recombination events (*R*_m_) ranged from 0 to 1 in both species across the nine genes (Table 3). Both STUCTURE and Neighbor-joining tree analyses of all samples identified two well-delimited genetic groups (Figures 4C,D). Similarly, AMOVA detected higher differentiation between species than between intraspecific populations (Table 2).

### Gene flow analysis by IMa2

**Figure 6:**
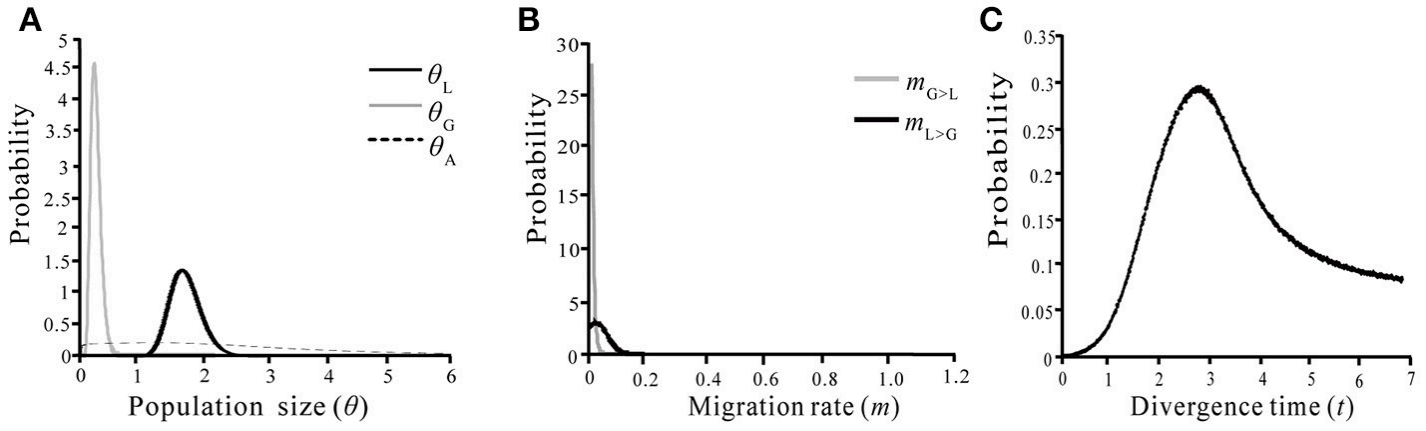
Marginal distributions of posterior probabilities of six demographic parameters estimated by the IM model. **(A)** Population size of *S. limprichtiana* (θ_*L*_), *S. grandifolia* (θ_*G*_) and ancestral species (θ_*A*_); **(B)** Migration rate between *S. limprichtiana* and *S. grandifolia*; **(C)** Divergence time of *S. limprichtiana* and *S. grandifolia*.

We used IMa2 to examine marginal posterior distributions of the probabilities of the six demographic parameters (*T, m*_GL_, *m*_LG_, θ_A_, θ_L_, and θ_G_) of two species under different models based on sequence variations of the nine nuclear loci (Table 4). All independent runs in the M-model gave consistent maximum likelihood estimates (MLEs) (Table 4, Figure 6), suggesting that the model has high reliability for the considered material. The MLEs indicate that interspecific divergence involved limited migration (*m*_LG_ = 0.068) from *S. grandifolia* to *S. limprichtian*, and more limited migration (*m*_GL_ = 0.004) in the opposite direction. Therefore, gene flow between *S. grandifolia* and *S. limprichtian* appears to have been very limited and asymmetrical. The marginal posterior densities of the divergence parameter according to MLEs, *t*, showed a sharp peak at 2.825, corresponding to a divergence time of *c*. 534,000 years, with a 95% highest posterior density (HPD) interval of *c*. 249,000–1,282,000 years. A much larger effective population size was derived for *S. limprichtiana* (*c*. θ_1_ = 1.74, *N*_1_ = 82,200 individuals, 95% HPD interval: *c*. 57,600–116,000 individuals) than for *S. grandifolia* (*c*. θ_2_ = 0.25, *N*_2_ = 11,900 individuals, 95% HPD interval: *c*. 5,100–22,900 individuals). The estimated effective population size of two species' ancestral population is c. θ_A_ = 1.60, *N*_A_ = 75,000 individuals (95% HPD interval: *c*. 56,700–330,000 individuals), smaller than that of *S. limprichtiana*, but clearly larger than that of *S. grandifolia* (Table 4).

**Table 4:** MLEs and the 95% HPD intervals of demographic parameters.

	**θ_1_**	**θ_2_**	**θ_A_**	**N1**	**N2**	**NA**	***m*_GL_**	***m*_LG_**	**2*N*_1_*m*_1_**	**2*N*_2_*m*_2_**	***t***	***T* (year)**
**L-MODE**												
MLE	1.748	0.252	1.588	8.25E+04	1.19E+04	7.50E+04	0.004	0.068	0.000344	0.0146	2.635	498,000
HPD95Lo	1.22	0.108	0	5.76E+04	5.10E+03	5.67E+02	0	0	0	0	1.319	249,000
HPD95Hi	2.46	0.484	6.988	1.16E+05	2.29E+04	3.30E+05	0.116	0.468	0.08634	0.0626	6.947	1,312,000
**M-MODE1**												
MLE	1.74	0.252	1.604	8.22E+04	1.19E+04	7.57E+04	0.004	0.068	0.000344	0.01454	2.825	534,000
HPD95Lo	1.22	0.108	0.012	5.76E+04	5.10E+03	5.67E+02	0	0	0	0	1.319	249,000
HPD95Hi	2.452	0.484	6.988	1.16E+05	2.29E+04	3.30E+05	0.116	0.468	0.08703	0.06311	6.787	1,282,000
**M-MODE2**												
MLE	1.74	0.252	1.604	82157.34	11898.65	75735.85	0.004	0.068	0.00036	0.01417	2.825	534,000
HPD95Lo	1.22	0.108	0.012	57604.57	5099.421	566.6024	0	0	0	0	1.319	249,000
HPD95Hi	2.452	0.484	6.988	115775.7	22852.96	329951.4	0.116	0.468	0.08676	0.06274	6.787	1,282,000
**M-MODE3**												
MLE	1.74	0.252	1.604	82157.34	11898.65	75735.85	0.004	0.068	0.00036	0.01417	2.825	534,000
HPD95Lo	1.22	0.108	0.012	57604.57	5099.421	566.6024	0	0	0	0	1.319	249,000
HPD95Hi	2.452	0.484	6.988	115775.7	22852.96	329951.4	0.116	0.468	0.08676	0.06274	6.787	1,282,000

*MLE, Maximum Likelihood Estimation; HPD, highest probablity density. θ_1_, θ_2_, and θ_A_ represent the effective diversity in S. limprichtiana, S. grandifolia, and the ancestral population, respectively; N_1_, N_2_, and N_A_ represent the effective size of population in S. limprichtiana, S. grandifolia, and the ancestral population, respectively; m_GL_ and m_LG_ represent the migration rate from the S. limprichtiana to the S. grandifolia and from the S. grandifolia to the S. limprichtiana, respectively; t and T indicate the scaled divergence time and the time of divergence, respectively. The results of L-mode and three independent M-mode runs are shown*.

## Discussion

We quantified morphological divergence and reproductive isolations between *S. limprichtiana* and *S. grandifolia*. In addition to the morphological differences mainly in the presence/absence of hairs recognized previously (Zhou et al., 2014), we further found the stable morphological differentiation between two species in the leaf and flower sizes, and numbers of flowers. Reproductive isolations were well-established between two species. Two sets of population genetic data similarly recovered high interspecific differentiation and divergence. All of these findings suggested that both species are “good species” rather than partly differentiated “subspecies” (Zhou et al., 2014). However, the divergence between them were estimated to occur in the recent past, probably during the Pleistocene, when the climate oscillated and forests accordingly fragmented and coalesced in eastern Asia (Qian and Ricklefs, 2000; Yu et al., 2000; Qian et al., 2003). Both stochastic processes and habitat selection probably contributed to such a fast divergence and strong post-pollination isolation.

### Interspecific differentiation

Our common garden experiments (designed to exclude effects of environmental variables) suggest that *S. limprichtiana* and *S. grandifolia* have developed stable morphological differentiation, including differences in the presence/absence of hairs on their calyxes, leaf blades and stems, leaf and flower sizes, and numbers of flowers per individual (Figure 2, Figures S2, S3). The morphological differentiation between them is as clear as that between closely related species of other genera in the family Brassicaceae (Zhou et al., 2001). All analyses of population genetic data obtained from analyses of the SSRs and low-copy nuclear genes also clearly delimited two species (Figure 4), in accordance with previous studies based on fewer individuals but different genetic markers (Hu et al., 2015, 2016). All of these lines of evidence indicate that *S. limprichtiana* and *S. grandifolia* are sufficiently well-differentiated and delimited to be acknowledged as separate “good species” (Hu et al., 2015) rather than subspecies (Zhou et al., 2014), although the distinction between subspecies and species is always arbitrary (Liu, 2016). In addition, as both species have the similar genome sizes (Figure S1, Table S3), their interspecific divergence did not involve any change in ploidy.

### Reproductive isolation

We failed to recover the pre-pollination isolation between two species. Pre-pollination isolation mechanisms, for example, differences in flowering phenology, are believed to have played major roles in the divergence of closely related species, especially species co-occurring in the same sites (Coyne and Orr, 2004). Such pre-pollination isolation mechanisms may frequently arise from selective divergence upon secondary contact following the development of an initial reproductive barrier (Hendry, 2009; Schemske, 2010; Sobel et al., 2010; Nosil, 2012). However, in our common garden experiments, flowering periods of *S. limprichtiana* and *S. grandifolia* strongly overlapped (Figure 3A), although we cannot exclude the possibility that this and other pre-pollination reproductive barriers probably occur in natural habitats of two species. *S. limprichtiana* prefers dry rocky places, while *S. grandifolia* generally occurs in wet sites along streams (Zhou et al., 2014). Such habitat differentiation probably results in differences in closely related plants' local flowering periods and rates of germination and/or survival probability because of the differences in water availability (Favre et al., 2017).

In contrast to the overlapping flowering periods, our artificial crossing experiments suggested the presence of strong post-pollination isolation between *S. limprichtiana* and *S. grandifolia*. Interspecific crossing using individuals of either species as the female parents and the other as pollen donors consistently resulted in substantially lower fruit set than intra-specific crossing (Figure 3B). Such intrinsic barriers arise mainly from Bateson-Dobzhansky-Muller genetic incompatibility due to genetic drift and natural selection, which result in the fixation of different alleles, new mutations and chromosomal rearrangements at different loci (Schluter, 2009). Further comparative analysis of two species' genomes and other molecular functional analyses are also warranted, to identify genetic changes associated with the observed post-pollination.

### Pleistocene divergence

We found that two species have fixed differentiated alleles at seven of nine examined nuclear loci, similarly suggesting the fast divergence in most loci. The high divergence was also recovered based on the SSRs results, consistent with the results based on other markers reported before (Hu et al., 2015, 2016). However, we dated two species' divergence to within the Pleistocene (Figure 6, Table 4) although such calculations should be treated cautiously because of the no-fossil calibrated mutation rate and the sequence variation from the limited loci. In addition, we detected extremely low gene flow between two species based on the sequence variations of nine nuclear loci, much lower than rates detected between other pairs of species that diverged recently, especially during the Pleistocene (Ikeda et al., 2009, 2012; Wang et al., 2013; Han et al., 2016).

What drove the fast divergence, development of the strong reproductive isolation and low gene flow between these two species within such a relatively short time? Three non-exclusive factors may have contributed to the fast differentiation between them. First, evergreen forests during the Pleistocene climatic oscillations might have fragmented as temperatures decreased in eastern Asia (Qian and Ricklefs, 2000; Yu et al., 2000), and the ancestor of these two species probably started to diverge allopatrically. In fact, all specimen records and our field investigations suggest that these two species currently occur in allopatric locations. Second, *S. limprichtiana* prefers dry habitats while *S. grandifolia* occurs in the wet habitats along the stream. These contrasted habitats may have produced strong selection pressure on the differentiations of two species (Wang et al., 2017). Finally, the effective population sizes of both species are small. The small populations may have accelerated the fixation of the different alleles between two species although both species are outcrossing. However, elucidating the relative importance of genetic drift and natural selection in the fast divergence and development of post-pollination isolation between *S. limprichtiana* and *S. grandifolia* remains challenging.

Our case study partly supported the prediction, derived from previous floristic analyses (e.g., Qian and Ricklefs, 2000), that Pleistocene climate changes likely promoted the production of the numerous young endemic species in eastern Asia through allopatric divergence and demographic oscillations as suggested for other endemic species occurring there (e.g., Chiang et al., 2012; Ikeda et al., 2012; Mitsui and Setoguchi, 2012; Nakamura et al., 2012; Han et al., 2016; Ito et al., 2017; Wang et al., 2017). Although further detailed studies of endemic species' origins are needed, these climate changes may have strongly contributed to the high species diversity in eastern Asia (Calonje et al., 2013). This study also adds to the limited number of known examples of Pleistocene speciation in plants from different regions of the world (e.g., Martin-Bravo et al., 2010; Ikeda et al., 2012; Levsen et al., 2012; Wang et al., 2013, 2017; Han et al., 2016).

## Conclusion

*S. limprichtiana* and *S. grandifolia* are well-differentiated in morphological characters, including the presence/absence of hairs, the leaf and flower sizes, and numbers of flowers. We also found that reproductive isolations were well-established between them. Interspecific divergence is high based on both sets of population genetic data. This divergence was further dated within the Pleistocene. All of these findings add our understanding of the divergence and the origin of the endemic species in eastern Asia.

## Author contributions

LZ, TZ, and QH: conceived and designed this study; TZ and HH: collected the samples, performed crosses, and morphometric analyses in common garden experiments; TZ, HH, LF, HZ, and LZ: acquired and analyzed data for the study; TZ, LF, HH, LZ, and QH wrote the manuscript.

### Conflict of interest statement

The authors declare that the research was conducted in the absence of any commercial or financial relationships that could be construed as a potential conflict of interest.
