# Supplementary material for: Interspecific Divergence of Two Sinalliaria (Brassicaceae) Species in Eastern China
Source: Front Plant Sci. 2018 Jan 31;9:77. doi: 10.3389/fpls.2018.00077 (PMC5797776; doi:10.3389/fpls.2018.00077)
Supplement: Supplementary file 1 [file DataSheet1.DOCX]

Supplementary Material

**Morphological differentiation, reproductive isolation and genetic divergence of two** ***Sinalliaria* species in eastern China**

Lei Zhang ^†1^, Tingting Zeng^†1^, Huan Hu^1^, Liqiang Fan^1^, Honglei Zheng^1^, Zheng Du^2^, Quanjun Hu*^1^

^1^MOE Key Laboratory for Bio-Resources and Eco-Environment, College of Life Science, Sichuan University, Chengdu, China

^2^National Supercomputing Center in Shenzhen

^†^These authors contributed equally to this work.

***Correspondence**:

Corresponding Author

Quanjun Hu

[huquanjun@gmail.com](mailto:huquanjun@gmail.com).

# Supplementary Tables

**Table S1.** Seed of two *Sinalliaria* species used for the common garden observation and experiments.

| Species | Populations | Individuals | Collection site | Longtitude (E) | Latitude (N) | Altitude (m) |
| --- | --- | --- | --- | --- | --- | --- |
| *S. grandifolia* | | | | | | |
|  | LJQ-HH-2014005 | 5 | Xiejiawu, Zhejiang, China | 118.94062 | 30.13779 | 334 |
|  | LJQ-HH-2014006 | 5 | Dashimen, Zhejiang, China | 118.91778 | 30.13792 | 278 |
| *S. limprichtiana* | | | | | | |
|  | LJQ-HH-2014002 | 5 | Tuankou, Zhejiang, China | 119.16522 | 30.01235 | 214 |
|  | LJQ-HH-2014007 | 5 | Wushan, Zhejiang, China | 120.16084 | 30.23713 | 70 |
|  | LJQ-HH-2015001 | 5 | Chaoshan,Zhejiang,China | 120.20732 | 30.43667 | 251 |

**Table S2.** Sampling locations of two *Sinalliaria* species (38 *S. limprichtiana* individuals and 36 *S. grandifolia* individuals) for population genetic analyses.

| Species | Populations | Individuals | Collection site | Longtitude (E) | Latitude (N) | Altitude (m) |
| --- | --- | --- | --- | --- | --- | --- |
| *S. grandifolia* | | | | | | |
|  | LJQ-HH-2013011 | 5 | Zhangshan, Anhui, China | 118.82142 | 30.11794 | 724~927 |
|  | LJQ-HH-2013012 | 7 | Zhangshan, Anhui, China | 118.79703 | 30.11861 | 676 |
|  | LJQ-HH-2014004 | 9 | Yunxiwu, Zhejiang, China | 118.90639 | 30.018056 | 879.5 |
|  | LJQ-HH-2014005 | 12 | Xiejiawu, Zhejiang, China | 118.94062 | 30.13779 | 334 |
|  | LJQ-HH-2014006 | 3 | Dashimen, Zhejiang, China | 118.91778 | 30.13792 | 278 |
| *S. limprichtiana* | | | | | | |
|  | LJQ-HH-2014001 | 8 | Changhua, Zhejiang, China | 119.2505 | 30.27642 | 361 |
|  | LJQ-HH-2014002 | 6 | Tuankou, Zhejiang, China | 119.16522 | 30.01235 | 214 |
|  | LJQ-HH-2014007 | 14 | Wushan, Zhejiang, China | 120.16084 | 30.23713 | 70 |
|  | LJQ-HH-2014008 | 10 | Yongjiaxian, Zhejiang, China | 120.83222 | 28.33013 | 364 |

**Table S3.** Comparison of frequency distributions of nuclear DNA contents in two *Sinalliaria* species obtained after mechanical isolation of nuclei and after protoplast lysis.

| Species | Individuals | 2C (pg) | Size of genome (Mb) | CV (%) |
| --- | --- | --- | --- | --- |
| *S. limprichtiana* | | | | |
|  | 1 | 1.556425 | 761.0916 | 2.17 |
|  | 2 | 1.548792 | 757.3594 | 1.24 |
|  | 3 | 1.70892 | 835.662 | 1.57 |
|  | Average | 1.604 ± 0.09 | 784.7043 | 1.66 ± 0.47 |
| *S. grandifolia* | | | | |
|  | 1 | 1.675758 | 819.4455 | 1.34 |
|  | 2 | 1.551724 | 758.7931 | 1.00 |
|  | 3 | 1.714019 | 838.1551 | 1.93 |
|  | Average | 1.647 ± 0.08 | 805.4646 | 1.42 ± 0.47 |

**Table S4.** The genetic diversity statistics and *F*-Statistics of 12 SSR loci for two *Sinalliaria* species

|  |  |  |  |  |  | *F*-Statistics | | |
| --- | --- | --- | --- | --- | --- | --- | --- | --- |
| Loci | *I* | *H*_o_ | *H*_e_ | *uH*_e_ | *F* | *F*_IS_ | *F*_IT_ | *F*_ST_ |
| SSR_1 | 1.058 | 0.371 | 0.591 | 0.658 | 0.35 | 0.371 | 0.552 | 0.287 |
| SSR_2 | 0.462 | 0.26 | 0.265 | 0.291 | 0.056 | 0.019 | 0.587 | 0.579 |
| SSR_3 | 0.836 | 0.225 | 0.476 | 0.514 | 0.626 | 0.527 | 0.718 | 0.403 |
| SSR_4 | 0.382 | 0.168 | 0.23 | 0.245 | 0.339 | 0.271 | 0.537 | 0.365 |
| SSR_5 | 0.687 | 0.377 | 0.423 | 0.462 | 0.097 | 0.109 | 0.404 | 0.331 |
| SSR_6 | 0.827 | 0.44 | 0.44 | 0.472 | 0.013 | 0.002 | 0.407 | 0.405 |
| SSR_7 | 0.159 | 0.112 | 0.093 | 0.102 | -0.154 | -0.204 | -0.044 | 0.133 |
| SSR_8 | 1.425 | 0.969 | 0.712 | 0.771 | -0.391 | -0.360 | -0.211 | 0.110 |
| SSR_9 | 0.791 | 0.344 | 0.443 | 0.482 | 0.222 | 0.223 | 0.576 | 0.454 |
| SSR_10 | 1.117 | 0.625 | 0.593 | 0.67 | -0.046 | -0.054 | 0.307 | 0.342 |
| SSR_11 | 0.448 | 0.097 | 0.221 | 0.237 | 0.507 | 0.563 | 0.846 | 0.647 |
| SSR_12 | 0.863 | 0.553 | 0.485 | 0.523 | -0.135 | -0.141 | 0.322 | 0.406 |
| Average | 0.754 | 0.378 | 0.414 | 0.452 | 0.124 | 0.111 | 0.417 | 0.372 |

*I*, Information Index; *H*_o_, observed Heterozygosity; *H*_e_, expected Heterozygosity; *u*H, unbiased expected heterozygosity; *F*, fixation Index.

# Supplementary Figures


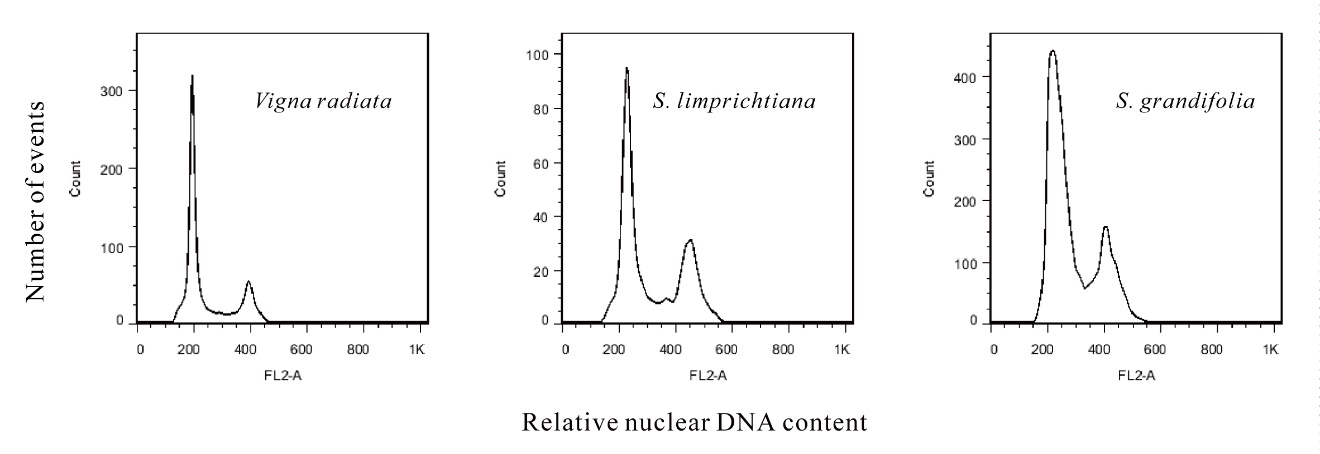


**Figure S1** Estimation of absolute nuclear DNA amount (genome size) for two *Sinalliaria* species. *Vigna radiata* served as internal reference standard (2C=1.4 pg DNA). The gain of the cytometer was adjusted so that the G1 peak of *Vigna* *radiata* was positioned on channel 200.


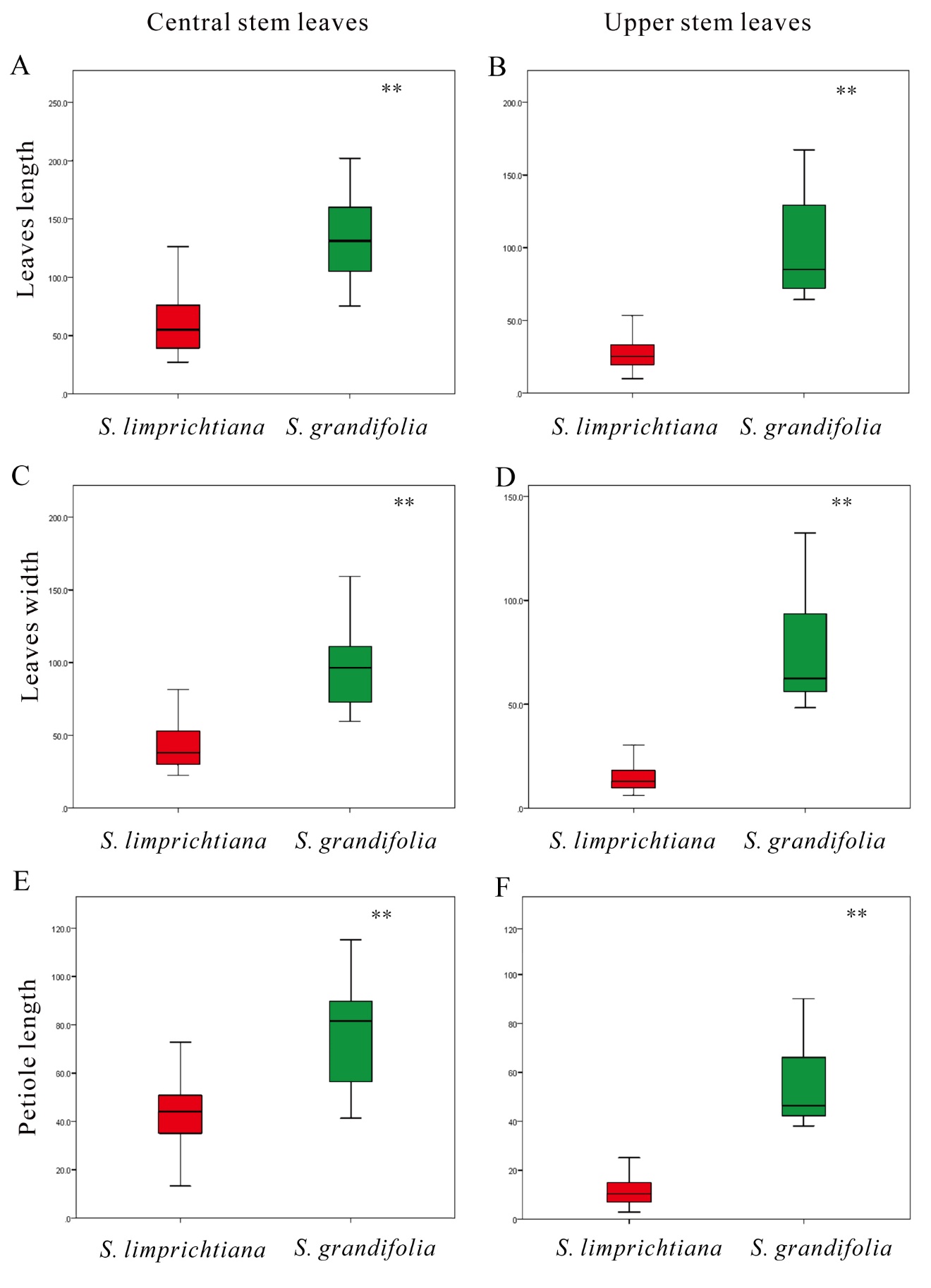


**Figure S2** Contrasted size of central stem leaves and upper stem leaves for two species (red and green boxes represent *S. limprichtiana* and *S. grandifolia* respectively; *P<0.05*).


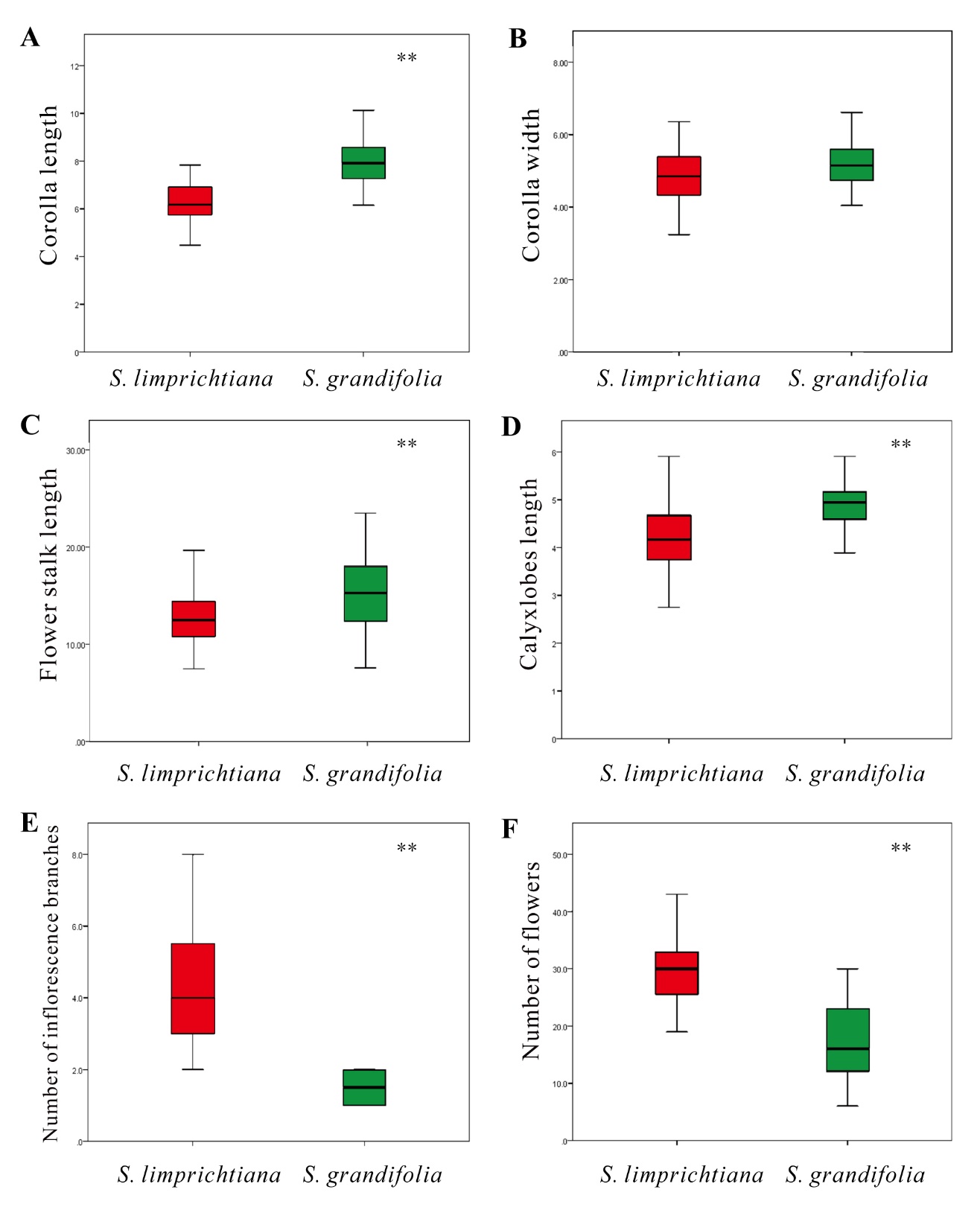


**Figure S3** Flower sizes of two species (red and green boxes represent *S. limprichtiana* and *S. grandifolia* respectively; *P<0.05*).


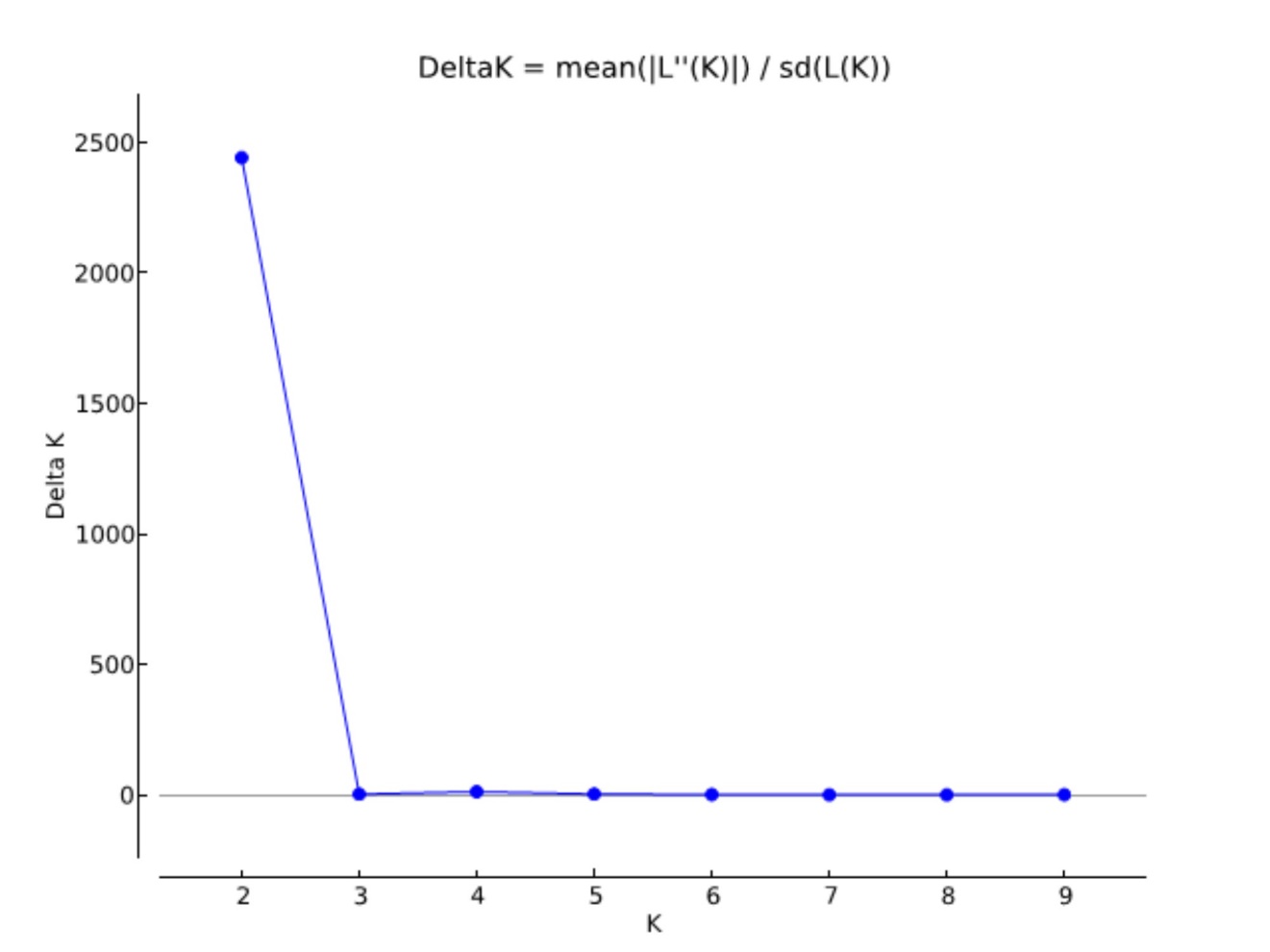


**Figure S4** Identification of the most suitable *K* values. The method of delta *K* was used to identify the accurate clusters in the population.
